# Carbon emission reduction in cement production catalyzed by steel solid waste

**DOI:** 10.1093/nsr/nwaf109

**Published:** 2025-03-27

**Authors:** Zhenggang Liu, Rui Lu, Yanfu Ma, Yue Hu, Xiaofei Zhang, Yanqing Wang, Wanjun Li, Lan Wang, Mansheng Chu, Rui Cai, Fang Lu, Zhongmin Liu

**Affiliations:** Dalian Institute of Chemical Physics, Chinese Academy of Sciences, China; Dalian Institute of Chemical Physics, Chinese Academy of Sciences, China; Dalian Institute of Chemical Physics, Chinese Academy of Sciences, China; Dalian Institute of Chemical Physics, Chinese Academy of Sciences, China; Dalian Institute of Chemical Physics, Chinese Academy of Sciences, China; Dalian Institute of Chemical Physics, Chinese Academy of Sciences, China; Dalian Institute of Chemical Physics, Chinese Academy of Sciences, China; China Building Materials Academy, China; School of Metallurgy, Northeastern University, China; Dalian Institute of Chemical Physics, Chinese Academy of Sciences, China; Dalian Institute of Chemical Physics, Chinese Academy of Sciences, China; Dalian Institute of Chemical Physics, Chinese Academy of Sciences, China

## Abstract

This study presents a novel method for reducing carbon emissions in cement production by using steel solid waste as a catalyst, offering a sustainable and cost-effective solution for environmental challenges.

Cement serves as the cornerstone of buildings and public infrastructure [1]. Over the course of approximately two centuries, the cement industry has undergone significant evolution and transformation [2]. However, its manufacturing process is characterized by intensive energy consumption and high carbon dioxide (CO_2_) emissions, accounting for an estimated 7.5% of global total emissions [3]. While alternative fuels can contribute to carbon emission reductions, the direct decarbonization technology employed in the carbonate decomposition process offers more efficiency [4–7]. Given the high-cost implications of incorporating nickel and ruthenium into cement products, it becomes imperative to segregate these metal catalysts from the clinker prior to its introduction into the rotary kiln [8,9]. The hurdle of harnessing cost-effective catalytic materials without necessitating their separation remains a formidable challenge. Herein, a pioneering strategy is developed that integrates carbon emission reduction in cement production with the utilization of steel solid waste. Specifically, iron-based catalysts, designed to mimic the composition of solid steel slag primarily consisting of Ca, Si, Al and Fe with trace Ni and Zn elements, were employed to catalyze the reaction of CaCO_3_ and CH_4_ for the co-production of CaO and syngas (Fig. 1a). Notably, the solid product necessitated no separation from the catalyst, thereby facilitating the direct manufacture of cement clinker with high iron content, as well as co-production of valuable chemicals and efficient utilization of industrial solid waste.

**Figure 1.**
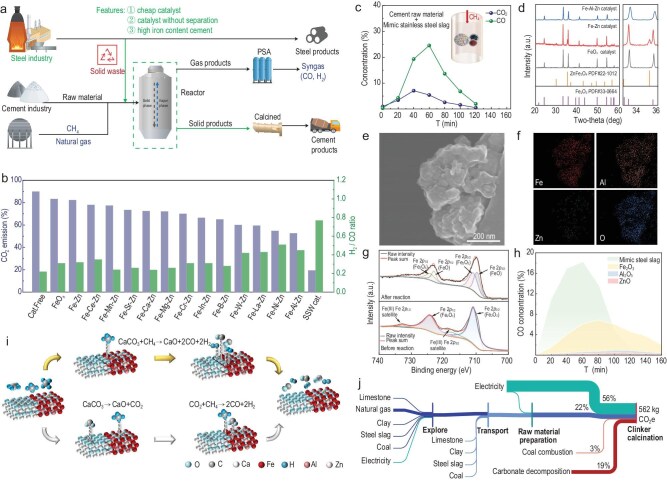
(a) Methane-integrated low-carbon cement production catalyzed by steel solid waste. (b) Effects of various Fe-based catalysts on the CO_2_ emissions and H_2_/CO ratio of the co-thermal conversion process of CaCO_3_ and CH_4_. Reaction conditions: 1.42 g CaCO_3_, 0.21 g Fe-based catalyst, 16 mL min^−1^ CH_4_, 800°C. SSW cat.: Steel Solid Waste catalyst. The ratio of Ni/CaCO_3_ was 0.75%. (c) Co-thermal conversion of commercial cement raw materials and CH_4_ over the synergy of SSW catalyst. Reaction conditions: 1.5 g raw materials, 0.3 g SSW catalyst, 16 mL min^−1^ CH_4_, 800°C. (d) XRD patterns for FeO_x_, Fe-Zn and Fe-Al-Zn catalyst. (e and f) STEM image of Fe-Al-Zn and corresponding energy dispersive X-ray spectroscopy (EDS) mapping. (g) XPS spectra of Fe 2*p* region in Fe-Al-Zn catalyst and used Fe-Al-Zn catalyst. (h) Comparison of catalytic performance on Fe_2_O_3_, ZnO, Al_2_O_3_ and Fe-Al-Zn catalysts. Reaction conditions: 1.64 g sample, 16 mL min^−1^ CH_4_, 800°C. (i) Possible reaction pathway of CH_4_ and CaCO_3_ over Fe-Al-Zn catalyst. (j) Life cycle assessment for reengineering of cement industry with SSW catalyst.

The experimental materials and catalysts undergo thorough mixing and subsequent granulation before being inserted into the fixed-bed reactor (Fig. S1). Upon thermal treatment at 800°C in a N_2_ atmosphere, CaCO_3_ decomposed into CaO with concomitant evolution of CO_2_. When the atmosphere was switched to CH_4_, the reaction evolved CO and H_2_ alongside CO_2_ as gaseous products (Fig. S2). Incorporation of Fe-based catalysts, such as FeO_x_, Fe-Zn, and Fe-Al-Zn composite systems, significantly enhanced the reaction process, yielding substantially elevated concentrations of CO and H_2_. Compared to FeO_x_ and Fe-Zn catalysts, the Fe-Al-Zn catalyst with three metal elements achieved the highest CO concentration, at ∼18.5%. Based on the Fe-Zn bimetallic system, other third metals were also introduced and their performance is compared with that of the Fe-Al-Zn catalyst (Fig. 1b and Fig. S3). With the CO_2_ emission from the decomposition of CaCO_3_ in N_2_ atmosphere set as the baseline at 100%, it dropped to 55% as the Fe-Al-Zn catalyst was used. A composite system labeled as Steel Solid Waste catalyst (SSW cat.) was prepared to simulate the composition of solid steel slag. The CO_2_ emission could be further reduced to 19.4% and the relatively higher H_2_/CO ratio in the gaseous product was advantageous for the subsequent utilization of syngas (Fig. 1b). During tests conducted with commercial cement raw material, the presence of components like Fe_2_O_3_ and Al_2_O_3_ induced a self-catalytic effect in a CH_4_ atmosphere, thereby facilitating the reaction between CaCO_3_ and CH_4_ to produce CO and H_2_ (Fig. S4a). Moreover, the amount of CO obtained was increased remarkably by using a Fe-Al-Zn and SSW catalyst (Fig. S4b and Fig. 1c).

The X-ray diffraction (XRD) patterns revealed the crystal phase structures of FeO_x_, Fe-Zn and Fe-Al-Zn catalysts (Fig. 1d). The main diffraction peaks of the FeO_x_ sample, such as 24.1^o^, 33.1^o^, 35.6^o^, 40.8^o^, 49.5^o^ and 54.1^o^, were assigned to Fe_2_O_3_. After the second element of Zn was introduced, Fe-Zn catalyst exhibited diffraction peaks corresponding to ZnFe_2_O_4_ alongside characteristic Fe_2_O_3_ reflections. With the further addition of Al element, the width of the half-peak of Fe_2_O_3_ became progressively larger, implying that Al was doped into the framework and the formed Al_2_O_3_ phase exists in an amorphous form. As revealed by scanning electron microscopy (SEM), the surface morphology of the catalysts changed significantly after the addition of Zn and Al, from tightly packed to loosely aggregated particles (Fig. S5). And the Brunauer–Emmett–Teller (BET) surface areas of FeO_x_, Fe-Zn and Fe-Al-Zn catalysts also increased gradually from 3.2 m^2^·g^−1^ to 31.6 m^2^·g^−1^ (Fig. S6). The scanning transmission electron microscope (STEM) image revealed an irregular morphology of the Fe-Al-Zn catalyst, while energy-dispersive X-ray spectroscopy (EDS) analysis demonstrated homogeneous dispersion of Fe, Al, Zn and O throughout the catalyst (Fig. 1e and f). The analysis result of inductively coupled plasma optical emission spectrometry (ICP-OES) reveals that the contents of Fe, Al and Zn in the catalyst were 51.1 wt%, 8.1 wt% and 4.9 wt%, respectively.

After in-depth analysis of a series of X-ray photoelectron spectroscopy (XPS) results, it was possible that the electronic state of Fe in the Fe-Al-Zn catalyst was influenced by the combined synergistic effect of Al and Zn. Specially, the electron transfer in the Fe-Al-Zn catalyst was probably from Al to Zn and Fe compared with the bimetallic Fe-based catalysts (Figs S7–S10 and Table S1). The O 1*s* spectrum of the Fe-Al-Zn catalyst exhibited two distinct peaks at 531.2 eV and 529.9 eV. And the peak at 531.2 eV was interpreted as the combined contribution from lattice oxygen of Al_2_O_3_ and oxygen vacancies, while the peak at 529.9 eV was assigned to lattice oxygen in metal oxides. Notably, the area ratio of these two peaks was 2.5/1, indicative of a surface enrichment of Al_2_O_3_. The integration of ICP, SEM and BET results further demonstrated that the enrichment of Al_2_O_3_ in the Fe-Al-Zn catalyst increased the specific surface area and dispersion of catalytic active sites. These changes optimized the microenvironment around the Fe sites, thereby promoting the reaction between CH_4_ and CaCO_3_. Two new peaks at 709.6 eV and 723.1 eV assigned to Fe^2+^ species appeared in the used Fe-Al-Zn catalyst, which might be attributed to the presence of reducing gases, such as H_2_ and CO (Fig. 1g). As the additive for cement, this change of Fe species could be negligible. The SSW catalyst was evaluated and according to the XPS result, Ni was mainly in the oxidized state (Fig. S11).

To identify the real active site in a Fe-Al-Zn catalyst, three individual metal oxides in equal amounts were used to evaluate catalytic performance under the same conditions. Compared to Al_2_O_3_ and ZnO, Fe_2_O_3_ exhibited an optimal catalytic performance, yielding a CO concentration of 7.5% (Fig. 1h and Fig. S12), implying that the iron center was the possible active site. The metallic iron catalyst (Fe^0+^) possessed the lowest volumetric ratio of product hydrogen H₂/CO as illustrated in Fig. S13, which was speculated to be caused by the high selectivity for the reverse water gas reaction [10]. Notably, the CO concentration on the Fe-Al-Zn catalyst was more than twice that of Fe_2_O_3_, further indicating the existence of a synergistic effect between Fe, Al and Zn. The Fe-Al-Zn catalyst was evaluated in the reaction of CO_2_ and CH_4_, and the formation of CO and H_2_ shows that the decomposition of CaCO_3_ followed by dry reforming might coexist with a direct reacting pathway (Fig. S14). Here, the value of k was defined as the variation of CO concentration per unit time, which reflected the reaction rate. It was clear that k values were 1.02 and 0.32 in 40 min, respectively. The primary reaction pathway was identified as the co-thermal conversion of CaCO_3_ under CH_4_ atmosphere. Thus, a plausible reaction mechanism was proposed and depicted in Fig. 1i. In the direct reaction pathway, the adsorbed CH_4_ interacted with the carbon-oxygen linkage at the Ca–Fe interface, subsequently converting into CO and H_2_ (upper route). The decomposition-adsorption pathway disclosed that CaCO_3_ initially decomposed to form CaO and CO_2_, which was then adsorbed and reacted with activated CH_4_ at the Ca–Fe interface to yield CO and H_2_ (lower route). Although both reaction pathways might occur simultaneously, the direct reaction between CH_4_ and CaCO_3_ was considered to be the dominant one. The dual-pathway mechanism accounted for the high CO concentration achieved with the Fe-Al-Zn catalyst.

Life cycle assessment (LCA) methodology was applied to evaluate the carbon emissions associated with simulative cement production. As shown in Fig. S15a, three scenario analyses were selected for energy consumption during the calcination phase, which involved conventional cement calcination utilizing coal combustion (1), cement calcination employing the innovative CH_4_ atmosphere furnace technology (2), and a prospective scenario applying the novel process with completely green electricity supply (3). In the process, the energy consumption of the CH_4_ atmosphere furnace was supplied by electricity, replacing 60% of the total coal consumption. In addition, the thermal CO_2_ generated from coal combustion in the rotary kiln could undergo a dry reforming reaction in the CH_4_ atmosphere furnace (Tables S2–S4). The traditional cement production process discharged approximately 918 kg of CO_2_ as a 1 ton clinker was produced (Fig. S15b). By using an electrically heated CH_4_ atmosphere kiln to replace the decomposition kiln, the carbon emissions from the production of a 1 ton clinker are reduced to approximately 562 kg, representing a direct reduction in CO_2_ emissions of over 38% (Fig. 1j). Considering a future scenario where the production was fully powered by green electricity (Fig. S15c), the new process would represent a reduction in carbon emissions of approximately 73%, indicating a significant potential for carbon reduction. Taking a cement clinker production line with an annual production capacity of 1 million tons as an example, after the low-carbon process reengineering, the cement output would remain unchanged, 190 million m^3^ of methane will be consumed, and about 740 million m^3^ of syngas could be produced as by-product, thereby increasing its sales revenue (Table S5).

In conclusion, the current research offers a reengineered strategy for the cement industry by incorporating synergies with steel solid waste exploration and presents a transformative approach to mitigating CO_2_ emissions. This work therefore lays a solid foundation for future research and implementation efforts aimed at decarbonizing the cement industry and contributing to global climate change mitigation efforts.

## Supplementary Material

nwaf109_Supplemental_File

## References

[1] Dunant CF, Joseph S, Prajapati R et al. Nature 2024; 629: 1055–61. 10.1038/s41586-024-07338-8 38778099 PMC11136652

[2] Schneider M, Romer M, Tschudin M et al. Cem Concr Res 2011; 41: 642–50. 10.1016/j.cemconres.2011.03.019

[3] Cheng D, Reiner DM, Yang F et al. Nat Commun 2023; 14: 8213. 10.1038/s41467-023-43660-x 38081830 PMC10713616

[4] Habert G, Miller SA, John VM et al. Nat Rev Earth Environ 2020; 1: 559–73. 10.1038/s43017-020-0093-3

[5] Zhang Y, Shen J, Zeng Y et al. Sep Purif Technol 2025; 354: 128816. 10.1016/j.seppur.2024.128816

[6] Xue Z, Guo J, Wu S et al. Sci China Chem 2023; 66: 1201–10. 10.1007/s11426-022-1537-6

[7] Kim SM, Abdala PM, Broda M et al. ACS Catal 2018; 8: 2815–23. 10.1021/acscatal.7b03063

[8] Shao B, Zhu Y, Hu J et al. Chem Eng J 2024; 483: 149098. 10.1016/j.cej.2024.149098

[9] Yin Q, Shen T, Li J et al. Chem Eng J 2023; 470: 144416.

[10] Xu M, Cao C, Xu J. Appl Catal A-Gen 2022; 641: 118682. 10.1016/j.apcata.2022.118682

