# Supplementary material for: Carbon emission reduction in cement production catalyzed by steel solid waste
Source: Natl Sci Rev. 2025 Mar 27;12(5):nwaf109. doi: 10.1093/nsr/nwaf109 (PMC11992567; doi:10.1093/nsr/nwaf109)
Supplement: nwaf109_Supplemental_File [file nwaf109_supplemental_file.pdf]

Supplementary Information

Carbon emission reduction in cement production catalyzed by steel solid waste

Zhenggang Liu[1,†], Rui Lu[1,†], Yanfu Ma[1], Yue Hu[1], Xiaofei Zhang[1], Yanqing Wang[1], Wanjun Li[1], Lan Wang[2], Mansheng Chu[3], Rui Cai[1,*], Fang Lu[1,*] and Zhongmin Liu[1]

[1]Dalian Institute of Chemical Physics, Chinese Academy of Sciences, China; [2]China Building Materials Academy, China; [3]School of Metallurgy, Northeastern University, China

*Corresponding authors. E-mails: cairui@dicp.ac.cn; lufang@dicp.ac.cn

[†]Equally contributed to this work.

1. Materials and Methods

Chemicals and materials

All the chemicals were used without further purification. $CaCO_3$ (AR), ZnO (99.7%), $Cr(NO_3)_3\ 9H_2O$ (AR), $Ni(NO_3)_2\ 6H_2O$ (99%), $Sr(NO_3)_2 \cdot 4H_2O$ (AR), Fe_2O_3(AR), ammonia (AR) were obtained from Tianjin Kemiou Chemical Reagent Co., Ltd. $Fe(NO_3)_3\ 9H_2O$ (AR), $Mg(NO_3)_2\ 6H_2O$ (AR), $Ca(NO_3)_2 \cdot 4H_2O$ (AR), Al_2O_3 (AR), H_3BO_3 (AR) were purchased from Sinopharm Chemical Reagent Co., Ltd. $Al(NO_3)_3\ 9H_2O$ (AR), $ZnCl_2$ (AR) were obtained from Damao Chemical Reagent Factory. $Zn(NO_3)_2\ 6H_2O$ (99.5%), $In(NO_3)_3 \cdot H_2O$ (99.5%), $Mn(NO_3)_3 \cdot 4H_2O$ (98%), $La(NO_3)_3\ 6H_2O$ (99%), WCl_6 (99%), $Ce(NO_3)_3\ 6H_2O$ (99.5%), Fe_3O_4 (99%) were obtained from Shanghai Aladdin Biochemical Technology Co., Ltd. Iron powder (99%) was obtained from Shanghai Macklin Biochemical Technology Co., Ltd.

The primary chemical constituents of the cement raw material were obtained from China National Building Material Group Southern Cement Company and determined by X-Ray Fluorescence (XRF). Upon converting to weight percentage based on the elemental composition, the composition was 75.2 wt% for $CaCO_3$, 13.0 wt% for SiO_2, 4.6 wt% for $MgCO_3$, 3.3 wt% for Al_2O_3, 2.4 wt% for Fe_2O_3, 0.3 wt% for sulfurous compounds and 1.2 wt% for others.

Catalyst preparation

Steel slag was mimicked by a homogeneous co-precipitation method according to consists of Fe, Al and Zn elements [1]. Typically, the elaborate preparation procedure for Fe-Al-Zn was outlined as follows: A mixture comprising 227.90 g of $Fe(NO_3)_3\ 9H_2O$, 93.50 g of $Al(NO_3)_3\ 9H_2O$, 25.18 g of $Zn(NO_3)_2\ 6H_2O$, and 11.60 g of $ZnCl_2$ was introduced into 250 mL of deionized water. This mixture was then heated to 50 ℃ and vigorously stirred until all components were completely dissolved, resulting in a brick-red solution. Then, 2.00 g of potato starch was added to deionized water and heated to 70 ℃ to form a paste followed by pouring into the aforementioned mixed salt solution, stirring for 30 min and naturally cooling to room temperature. The obtained mixed salt solution and 500.00 g of dilute ammonia solution (pH=9.5) was pumped steadily into a beaker separately using two peristaltic pumps. Subsequently, the resulting mixture was vigorously stirred for 30 min and then

aged for 12 h. After washing and filtering for 3 times with dilute ammonia solution, the brick-red solid was dried at room temperature for 12 h and calcined in a muffle furnace to 650 ℃ with a heating rate of 10 ℃ min^{-1} in an air atmosphere and held for 10 h. The obtained sample was denoted as Fe-Al-Zn catalyst. The other metal doping preparation process was similar to that except for different amount of metal salts.

Steel solid wastes (SSW) were mimicked according to consists of Fe, Al, Zn and trace amounts of Ni elements. The Fe-Al-Zn catalysts were first prepared by the co-precipitation method as described previously. Thereafter, according $Ni(NO_3)_2$ $6H_2O$ was accurately weighed and transferred into a flask with 1.0 mL of deionized water. After dissolving, 1.00 g of nanometer ZnO was added with continuous stirring for 20 min and ultrasonic dispersion for another 20 min. The resulting mixture was left to rest for 24 h and dried at 110 ^oC for 12 h. After grinding, the solid powder was reduced in hydrogen atmosphere at 450 ^oC for 3 h and mixed with Fe-Al-Zn for grinding and granulation to obtain SSW catalysts.

The typical sample preparation process for catalyst performance evaluation was described below: 1.42 g of $CaCO_3$ and 0.21 g of Fe-Al-Zn catalyst were mixed and grinded. The mixture was pressed to form tablets and crushed followed by sieving to 20~40 mesh. After being dried at 110 ℃ for 30 min, the sample was loaded onto a fixed bed for performance evaluation.

Catalytic performance evaluation

A schematic diagram of the fixed bed reactor system was presented (Fig. S1). The system consists of three primary components: a gas control module, a reaction chamber, and a product analysis unit. In detail, the device is equipped with two mass flow controllers (MFC), a tube furnace and a gas chromatography (GC) analyzer. The stainless-steel reactor tube, with an inner diameter of 10 mm and a length of 700 mm, is equipped with thermocouples for precise temperature monitoring and an external heater to supply the requisite thermal energy for the reaction process.

The incoming flow rates of the feed gases are meticulously controlled by MFCs, while the outflow rates of the resulting gases are precisely measured utilizing a soap film flow meter. The evolution of the product gas composition over time is monitored using a TDX-01 column with a 20 min of sampling interval for gas analysis. The raw gases, CH_4 and N_2, are supplied from high purity steel cylinders and their flow rates

are precisely controlled by MFCs. The temperature within the tube furnace is precisely regulated by a programmable logic controller. Following condensation and subsequent removal of water vapor through a condenser, the product gases are directed to a GC for real-time compositional analysis. The conversion of $CaCO_3$ is subsequently determined by integrating the data obtained from GC analysis with the cumulative gas outlet flow rate.

During the experimental procedure, $CaCO_3$ powder was thoroughly mixed with the catalyst, followed by sieving to achieve a uniform particle size distribution within the specified range of 20~40 mesh. The prepared samples, in conjunction with quartz wool and quartz sand, were meticulously positioned within the isothermal zone of the reactor tube (Fig. S2). Typically, a precisely measured 1.64 g of the prepared sample was introduced into the reactor vessel before each experimental run. Nitrogen gas (99.999% purity) was used as a purge medium to expel any residual atmospheric gases from the tube reactor to reduce the risk of undesirable chemical reactions. The fixed bed reactor was gradually heated from ambient temperature to a target temperature of 800 ℃ at a controlled rate of 5 ℃ min^{-1}, where it was maintained for the duration of the reaction. Samples were taken at regular intervals of 20 min during the isothermal hold stage.

Characterization

Powder X-ray diffraction (XRD) was performed on a Rigaku D/Max 2500/PC diffractometer with Cu Kα radiation (λ = 0.15418nm) operated at 40 kV/200mA. The wide-angle patterns were recorded from 10° to 90° (2θ) at a scan rate of 2.5 ° min^{-1}. A scanning electron microscope (SEM, FEI Magellan 400 FEG), and a scanning transmission electron microscope (STEM, Hitachi HD-2700 and S5500) were used to characterize the morphology of catalyst. The contents of Fe, Al, Zn, and Ni in the catalyst were measured using a Horiba EMIA-8100 instrument. X-ray photoelectron spectroscopy (XPS) was determined by a Thermofisher Excalab X + spectrometer equipped with a monochromated aluminum source (Al Kα= 1486.6 eV). The binding energies were corrected with C 1s of 284.80 eV.

Products analysis

Analysis of gas and liquid products. Gas products were analyzed by two tandem GC equipped with thermal conductivity detector (TCD) and flame ionization detector (FID). Specifically, the analysis of H_2 was performed on an Agilent 7890A GC system using an Agilent 19095P-MS0 capillary column. The inlet temperature was set at 250 ℃ and the column was kept at 80 ℃ for 14 min. The carrier gas was argon with a flow rate of 30 mL min^{-1}. The analysis of CH_4, CO and CO_2 were performed on an Agilent 7890B GC system using a HayeSep Q capillary column and a MolSieve 5A with both TCD and FID detectors. The inlet temperature was set at 250 ℃. The column was initially held at 80 ℃ for 5 min, then heated to 160 ℃ at a rate of 20 ℃ min^{-1}, and kept for 5 min. The carrier gas was He with a flow rate of 30 mL min^{-1}. CH_4 was quantified on the Agilent 7890B GC system with FID detector. CO and CO_2 were quantified by TCD detector. The results obtained from GC analysis were the volume per centage of mixed gas and further transformed into the mole numbers of each component using the Ideal Gas Equation.

Comparing the CO_2 emitted during the experiment with the CO_2 that could be released from the theoretical decomposition of the input $CaCO_3$, the total CO_2 emission rate (α) was deduced. as described by Eq. (1).

$$\alpha\ (\%) = \frac{V_{out}}{V_{CO2}} \cdot 100\% \qquad (1)$$

α: the total CO_2 emission rate (%).

V_{out}: CO_2 that could be liberated from the $CaCO_3$ introduced into the experiment, quantified in milliliters (mL).

V_{CO2}: CO_2 that could be liberated from the $CaCO_3$ introduced into the experiment, quantified in milliliters (mL).

The conversion of $CaCO_3$ to non-CO was quantitatively determined by using the area under the chromatographic peaks in conjunction with the volumetric flow rates of both the inlet and outlet gases, as described by Eq. (2). The sampling procedure was performed systematically at 20-minute intervals, with the effluent gas flow rate meticulously recorded and documented. The exit concentration of CO_2 was accurately

determined at each time point based on the integrated area under the peak in the chromatogram. The average concentration between two consecutive sampling points was used to estimate the release of CO_2 for each measurement interval. This methodological approach ensured a comprehensive and scientifically rigorous analysis of the reaction kinetics and efficiency of the catalytic process.

$$V_{\text{out}} = \sum_{i=1}^{n-1} \frac{C_i + C_{i+1}}{2} \cdot \Delta t \cdot \frac{X_i + X_{i+1}}{2} \cdot 100\% \qquad (2)$$

C_i: The volumetric flow rate of gas at the outlet measured at the i-the sampling point, expressed in milliliters per minute (mL $\cdot$min^{-1}).

Δt_i: The temporal separation between successive sampling points, measured in minutes (min).

X_i: The volumetric percentage of CO_2 at the outlet gas measured at i-th sampling point (vol%).

The H_2/CO ratio of syngas is based on the ratio of H_2 and CO concentrations in the outlet gas corresponding to the highest CO_2 concentration measured during the reaction.

Quantification of product carbon footprint. Life Cycle Assessment (LCA) stands as a holistic analytical framework designed to evaluate the environmental performance of a product's life cycle. Typically, the carbon footprint of product is employed as an evaluative tool to measure the emission of greenhouse gases across the product's life cycle, thereby gauging its impact on global warming. In this study, LCA was used to evaluate the emissions of greenhouse gases (GHGs), including CO_2, CH_4, and nitrous oxide (N_2O), with the results articulated in terms of kilograms of CO_2 equivalents (kg CO_2e), which were calculated by multiplying the mass of the GHG released or removed by the 100-year Global Warming Potential (GWP) as provided by the Intergovernmental Panel on Climate Change (IPCC). This approach provided a unified measurement standard for comparing the relative and long-term environmental impact of different greenhouse gases on climate change. However, in certain situations, to more accurately evaluate and compare the carbon efficiency of different products or services, the carbon

footprint may be further refined to kilograms of CO_2 equivalents (kg CO_2e) per declared unit of product [2].

When using life cycle assessment for cement clinker, a cradle-to-gate analysis was considered with direct and indirect emissions associated with the main life cycle stages, such as raw material acquisition, transportation and clinker production [3]. Primary data, such as that for clinker production, were primarily based on factory processes and experimental procedures. Secondary data, which included information on raw material acquisition, transportation, and other related stages, were retrieved from databases in GREET Software and Gabi Software. The functional unit is one ton of clinker, which was the key ingredient of cement products. The carbon footprint of the product represented the cumulative climate change impact of all GHGs, as illustrated in Eq. (3).

$$E_{GHG}=\sum(AD_i \times EF_i \times GWP_i) \quad (3)$$

E_{GHG} represented the carbon footprint of a product, measured in kilograms of CO_2 equivalents (kg CO_2e)

AD_i referred to the activity data for the *i*-th greenhouse gas emission activity, with units determined based on the specific emission source.

EF_i was the emission factor for the *i*-th activity that generated greenhouse gas, with units corresponding to the units of the activity data.

GWP_i was the global warming potential (GWP) value of the greenhouse generated by *i*-th activity.

2. Supporting Figures

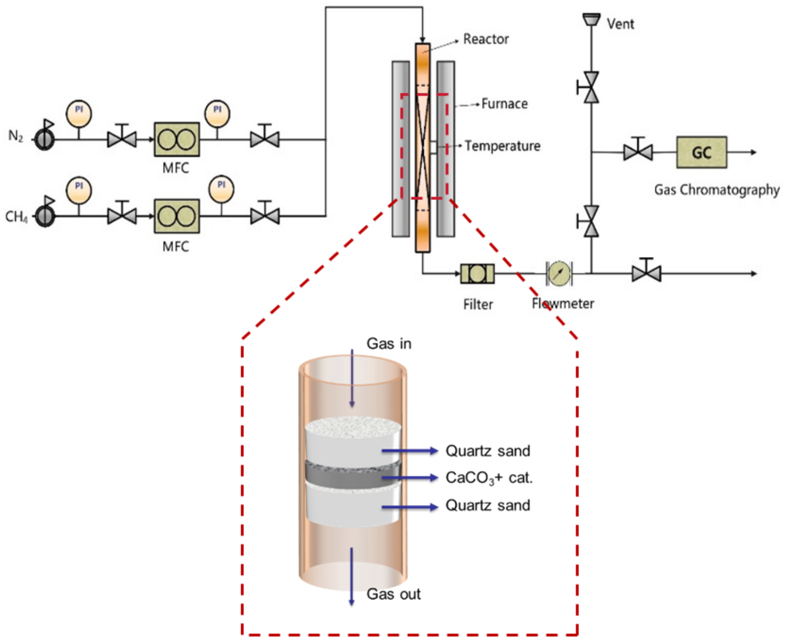

Fig. S1 Schematic of the experimental setup

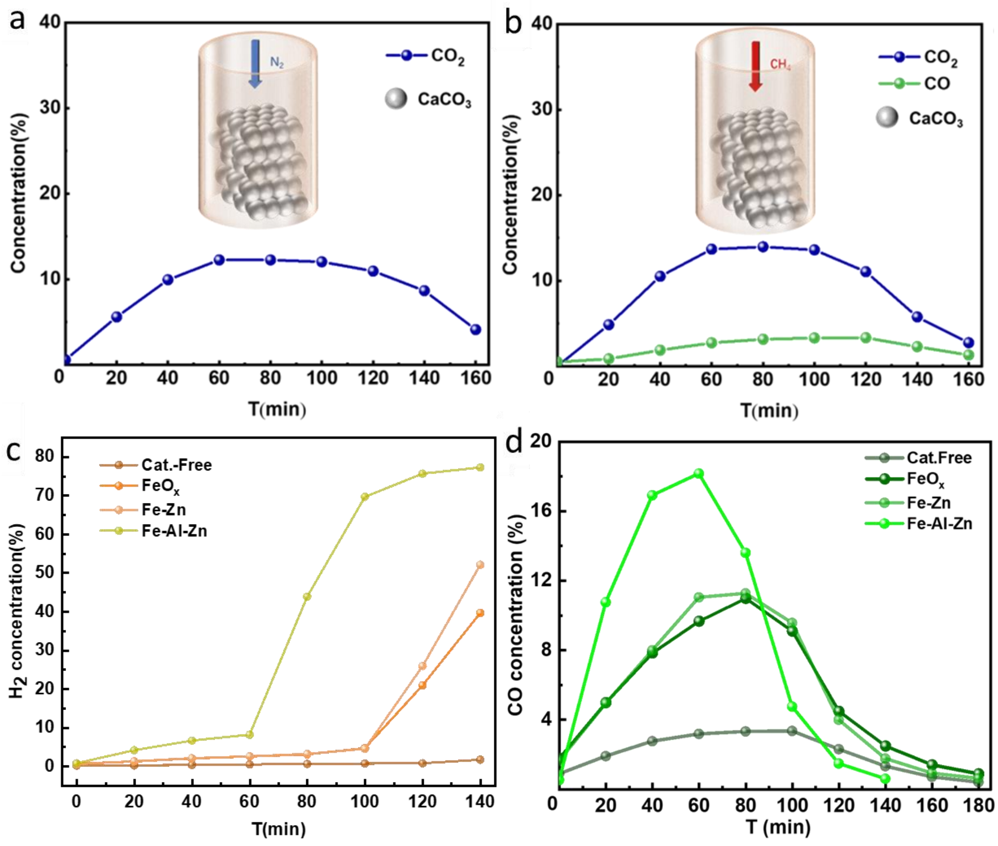

Fig. S2 (**a**) Decomposition of $CaCO_3$ in N_2 atmosphere. (**b**) Decomposition of $CaCO_3$ in CH_4 atmosphere. **(c)** Hydrogen concentration for different iron-based catalysts. (**d**) Carbon monoxide concentration for different iron-based catalysts. Reaction conditions: 1.42 g $CaCO_3$, 0.21 g Fe-based catalyst, 16 mL min^{-1} N_2 or CH_4, 800 ^oC.

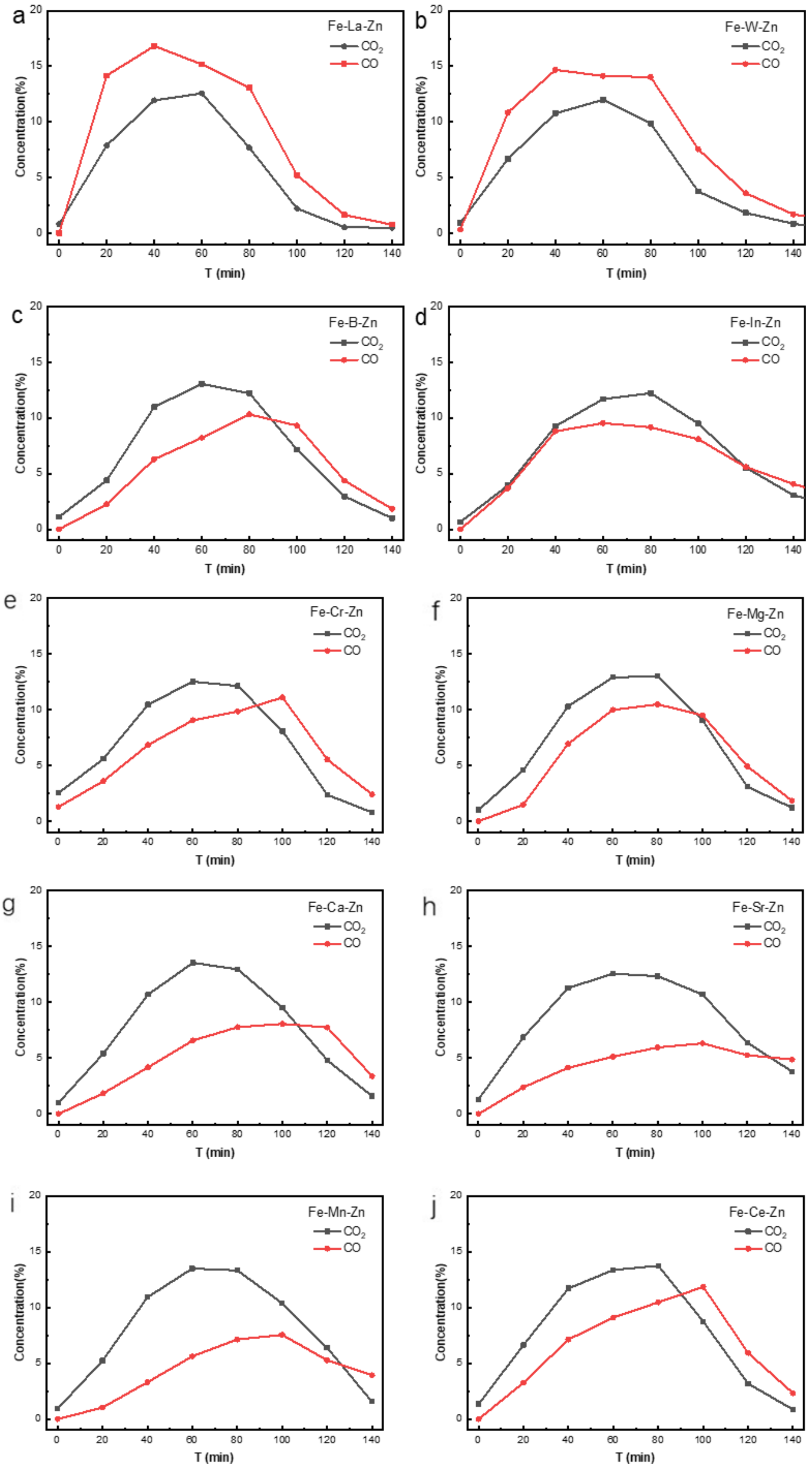

Fig. S3 Reaction of iron-based catalysts doped with different metals. (**a**) Fe-La-Zn. (**b**) Fe-W-Zn. (**c**) Fe-B-Zn. (**d**) Fe-In-Zn. (**e**) Fe-Cr-Zn; (**f**) Fe-Mg-Zn; (**g**) Fe-Ca-Zn; (**h**) Fe-Sr-Zn. (**i**) Fe-Mn-Zn. (**j**) Fe-Ce-Zn.Reaction condition: 1.42 g $CaCO_3$, 16 ml min^{-1} CH_4, 800 ^oC.

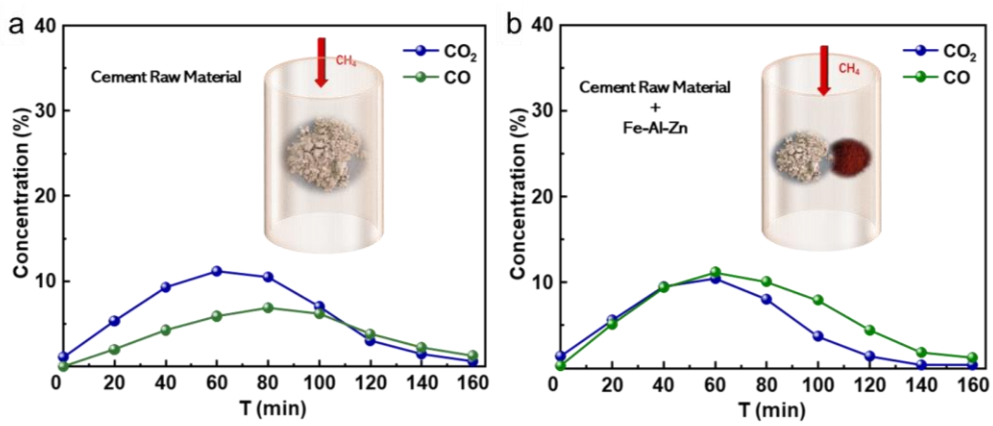

Fig. S4 (**a**) Decomposition of commercial cement raw materials in CH_4 atmosphere. (**b**) Co-thermal conversion of commercial cement raw materials and CH_4 over Fe-Al-Zn. Reaction condition: 1.5 g raw materials, 0.3g SSW catalyst, 16 mL min^{-1} CH_4, 800 ^oC.

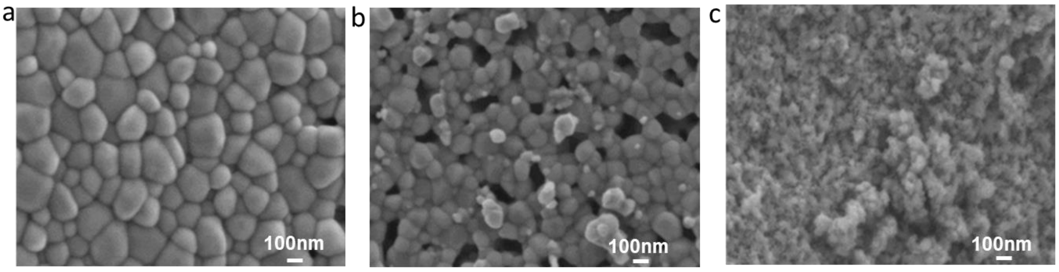

Fig. S5 SEM of typical catalysts (a) FeO_x. (b) Fe-Zn. (c) Fe-Al-Zn.

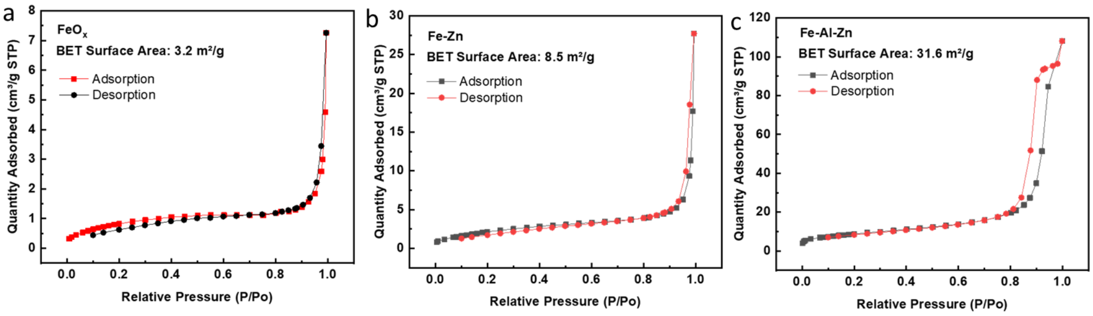

Fig. S6 BET of typical catalysts (**a**) FeO_x. (**b**) Fe-Zn. (**c**)Fe-Al-Zn.

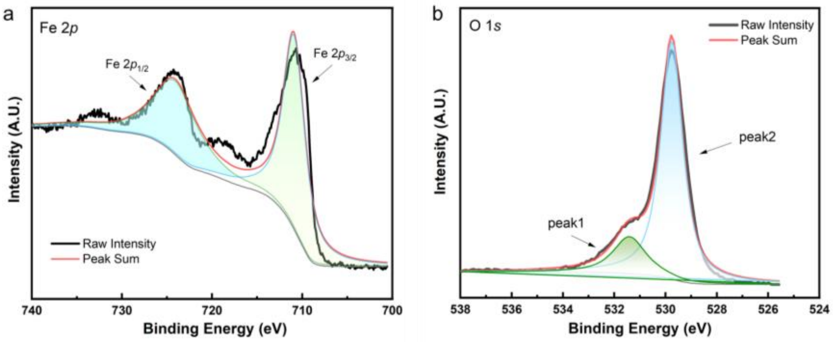

Fig. S7 XPS spectra of FeO_x catalyst. (**a**) Fe 2*p*; (**b**) O 1*s*.

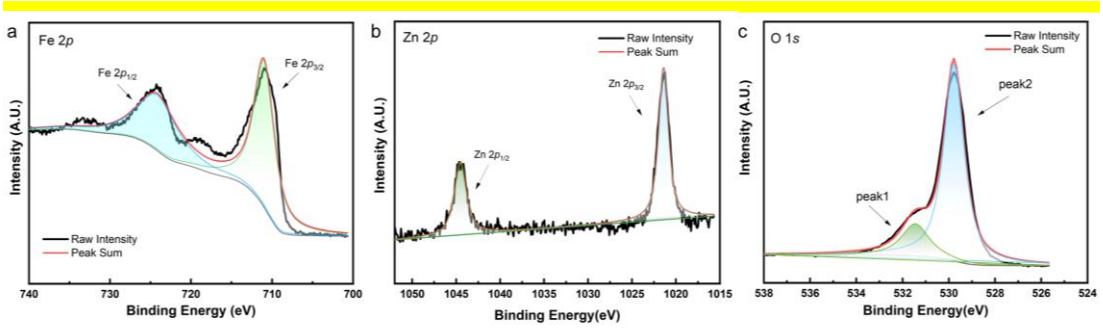

Fig. S8 XPS spectra of metal elements in Fe-Zn catalyst. (**a**) Fe 2*p*; (**b**) Zn 2*p*; (**c**) O 1*s*.

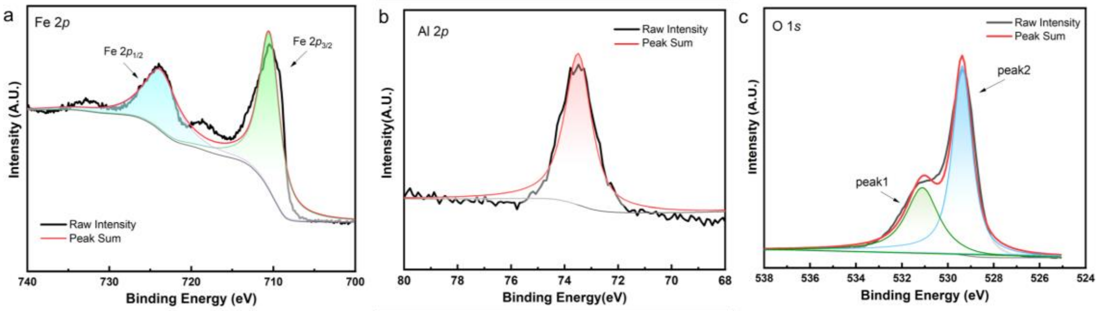

Fig. S9 XPS spectra of Fe-Al catalyst. (**a**) Fe 2*p*; (**b**) Al 2*p*; (**c**) O 1*s*.

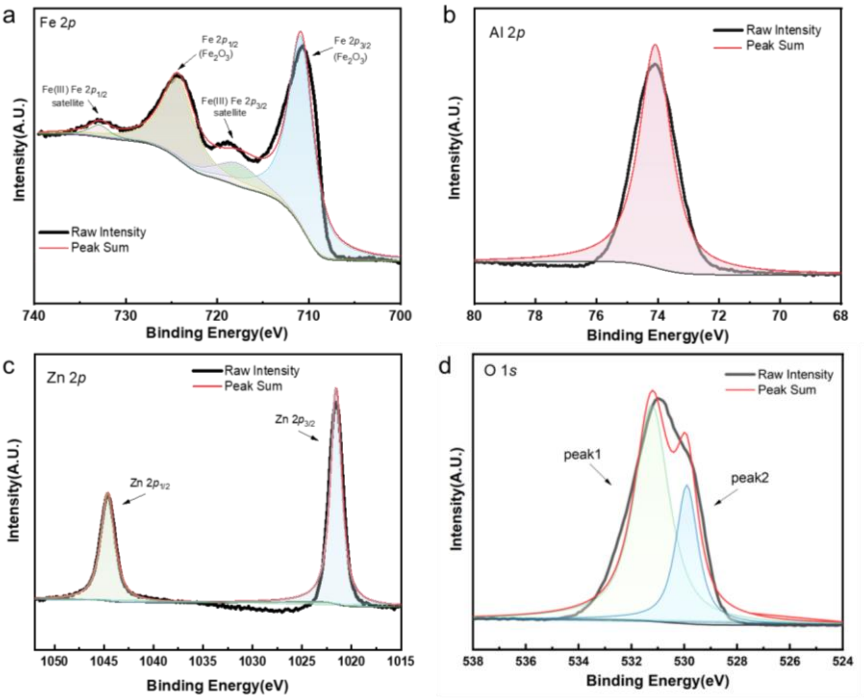

Fig. S10 XPS spectra of Fe-Al-Zn catalyst. (**a**) Fe 2*p*; (**b**) Al 2*p*; (**c**) Zn 2*p*; (**d**) O 1*s*.

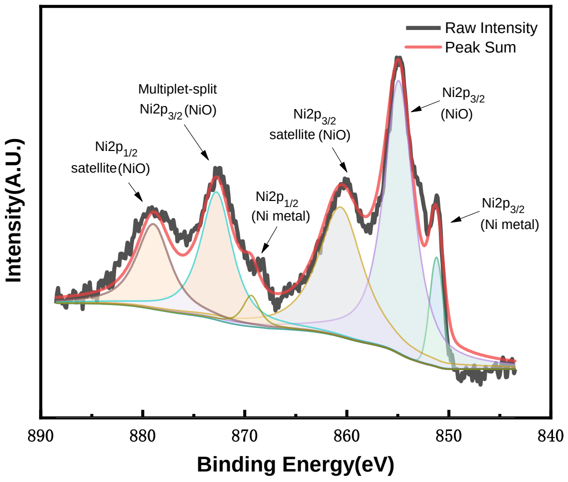

Fig. S11 XPS pattern for Ni in mimic steel solid waste catalyst.

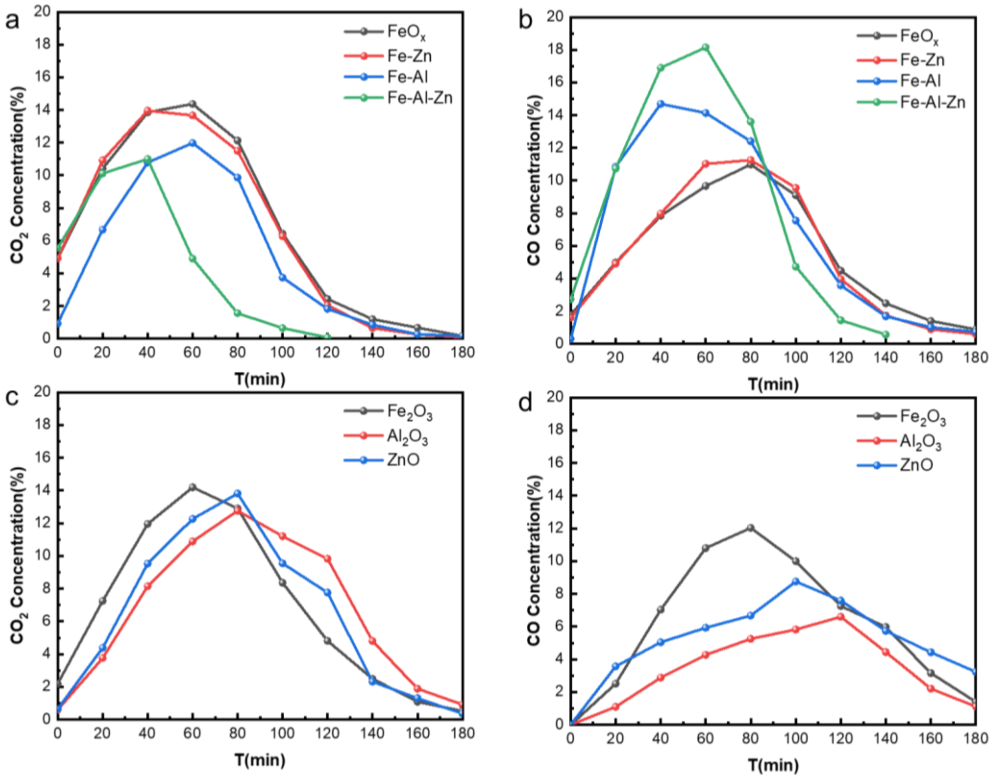

Fig. S12 Comparison of CO_2 and CO concentration variations in the reactions catalyzed by Fe-based catalysts and commercial metal oxides. (**a**) Variation in CO_2 concentration during the reaction with Fe-based catalysts prepared by co-precipitation method. (**b**) Variation in CO concentration during the reaction with Fe-based catalysts prepared by co-precipitation method. (**c**) Variation in CO_2 concentration during the reaction catalyzed by Fe_2O_3, Al_2O_3 and ZnO. (**d**) Variation in CO concentration during the reaction catalyzed by Fe_2O_3, Al_2O_3 and ZnO. Reaction conditions:1.42 g $CaCO_3$, 16 mL min^{-1} CH_4, 800 ^oC. The mass ratio of catalyst to $CaCO_3$ is 15%.

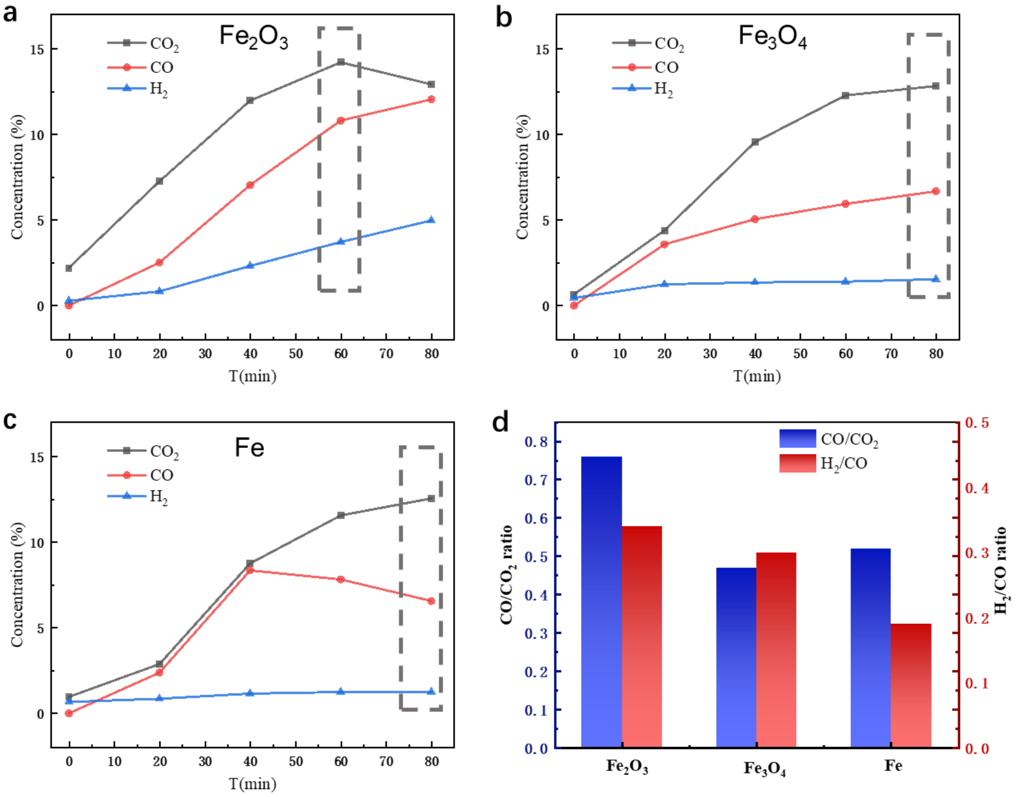

Fig. S13 Effect of valence in iron-based catalysts. (**a**) Fe_2O_3. (**b**) Fe_3O_4. (**c**) Fe metal. (**d**) Ratio of CO to CO_2 as well as ratio of H_2 to CO at the peak concentration of CO_2. Reaction condition: 1.42 g $CaCO_3$, 16 mL min^{-1} CH_4, 800 ^{o}C.

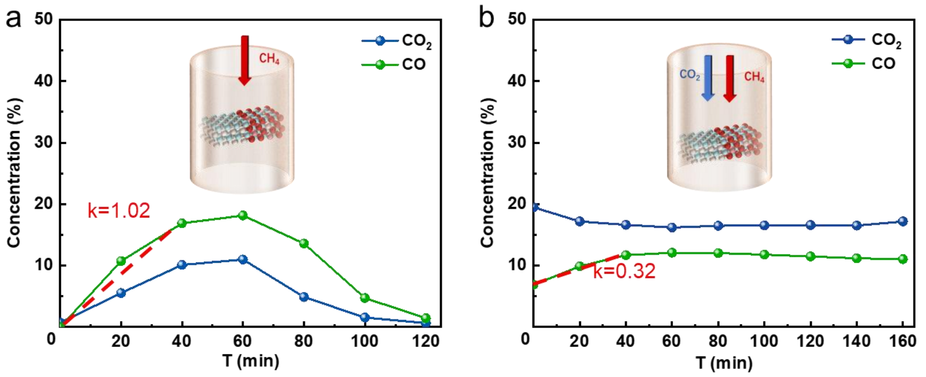

Fig. S14 (**a**) Co-thermal conversion of $CaCO_3$ and CH_4 over Fe-Al-Zn catalyst. Reaction condition:1.42 g $CaCO_3$, 0.21 g catalyst, 16 mL min^{-1} CH_4, 800 ^{o}C. (**b**) Catalytic performance of Fe-Al-Zn catalyst under CH_4 and CO_2 atmosphere. Reaction condition: 0.21 g catalyst, 6 mL min^{-1} CO_2, 800 ^{o}C. Define k as the variation of CO concentration per unit time.

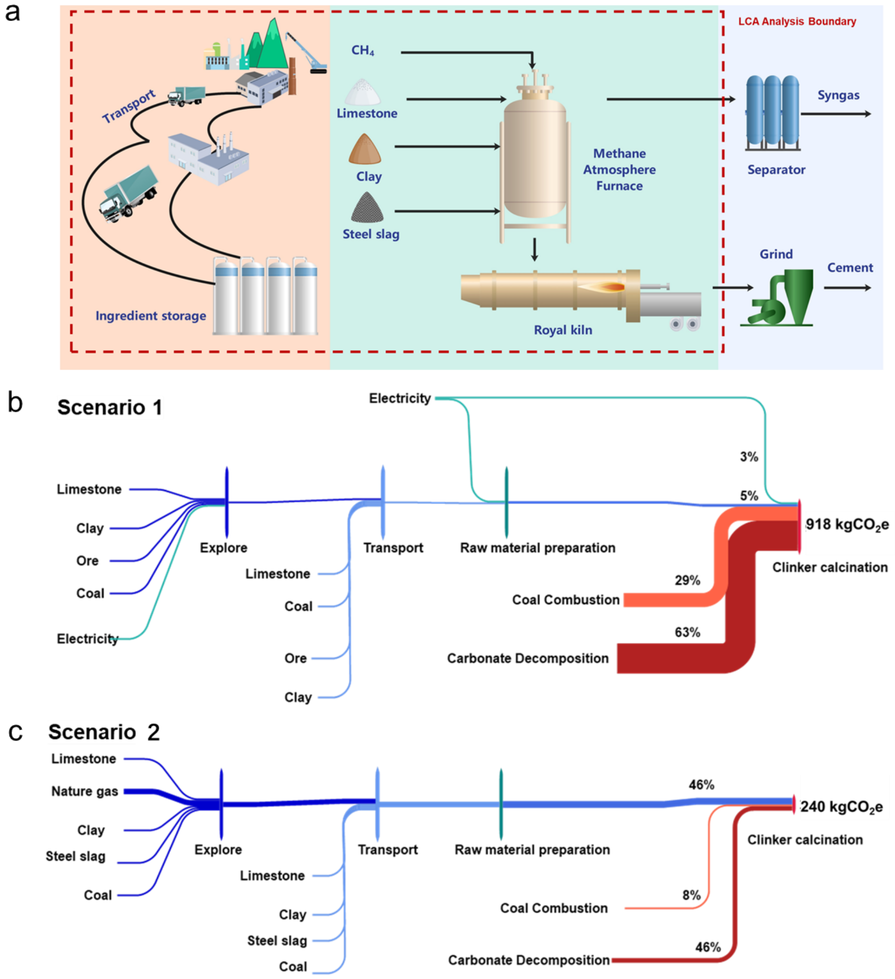

Fig. S15 (**a**) System boundary of life cycle assessment for reengineering of cement industry. The system boundaries include raw material extraction, raw material transportation, raw meal preparation, and clinker calcination. Entire life cycle of the three different scenarios: (**b**) Traditional process. (**c**) Future reengineering process with green electricity.

3. Supporting Tables

Table S1. Binding energies of elements in selected Fe-based catalyst

Spectral line	Catalyst	Orbitals	Binding energy/eV
Fe 2*p*	FeO_x	$2p_{3/2}$	710.9
		$2p_{1/2}$	724.0
	Fe-Zn	$2p_{3/2}$	710.9
		$2p_{1/2}$	724.1
	Fe-Al	$2p_{3/2}$	710.4
		$2p_{1/2}$	723.7
	Fe-Al-Zn	$2p_{3/2}$	710.7
		$2p_{1/2}$	723.9
Al 2*p*	Fe-Al	2*p*	73.5
	Fe-Al-Zn	2*p*	73.9
Zn 2*p*	Fe-Zn	$2p_{3/2}$	1021.4
		$2p_{1/2}$	1044.5
	Fe-Al-Zn	$2p_{3/1}$	1021.1
		$2p_{1/2}$	1044.3
O 1*s*	FeO_x	peak1	531.4
		peak2	529.7
	Fe-Zn	peak1	531.4
		peak2	529.8
	Fe-Al	peak1	531.1
		peak2	529.4
	Fe-Al-Zn	peak1	531.2
		peak2	529.9

Table S2. Life cycle inventory of Scenario 1

Inputs		Amount	Outputs	Amount
	**Raw meal preparation**			
Limestone (ton)		1.3223×10^{0}	Carbon dioxide (CO_2) (kg)	1.592×10^{1}
Clay (ton)		1.959×10^{-1}		
Ore (ton)		1.143×10^{-1}		
Coal average (ton)		1.026×10^{-1}		
Electricity (kwh)		4.89×10^{0}		
	**Transport**			
Road (km)		1×10^{2}	Carbon dioxide (CO_2) (kg)	1.294×10^{1}
Railway (km)		2×10^{2}		
	**Raw material preparation**			
Raw material (ton)		1.6325×10^{0}	Carbon dioxide (CO_2) (kg)	1.836×10^{1}
Coal average (ton)		1.026×10^{2}		
Electricity (kwh)		3.22×10^{1}		
	**Calcination**			
Raw material (ton)		1.6325×10^{0}	Carbon dioxide (CO_2) (kg)	8.707×10^{2}
Coal average (ton)		1.026×10^{2}	Clinker (ton)	1
Electricity (kwh)		2.8×10^{1}		

Table S3. Life cycle inventory of Scenario 2

Inputs	Amount	Outputs	Amount
**Raw meal preparation**			
Limestone (ton)	1.3223×10^{0}	Carbon dioxide (CO_2) (kg)	9.099×10^{1}
Clay (ton)	1.959×10^{-1}		
Steel slag (ton)	1.143×10^{-1}		
Coal average (ton)	4.104×10^{-2}		
Electricity (kwh)	4.89×10^{0}		
Natural gas (ton)	2.085×10^{2}		
**Transport**			
Road (km)	1×10^{2}	Carbon dioxide (CO_2) (kg)	1.312×10^{1}
Railway (km)	2×10^{2}		
Pipeline (km)	2×10^{2}		
**Raw material preparation**			
Raw material (ton)	1.6325×10^{0}	Carbon dioxide (CO_2) (kg)	1.836×10^{1}
Coal average (ton)	4.104×10^{-2}		
Electricity (kwh)	3.22×10^{1}		
**Calcination**			
Raw material (ton)	1.6325×10^{0}	Carbon dioxide (CO_2) (kg)	4.399×10^{2}
Coal average (ton)	4.104×10^{-2}		
Electricity (kwh)	5.01×10^{2}	Clinker (ton)	1
Natural gas (ton)	2.085×10^{2}		

Table S4. Life cycle inventory of Scenario 3

Inputs	Amount	Outputs	Amount
**Raw meal preparation**			
Limestone (ton)	1.3223×10^{0}	Carbon dioxide (CO_2) (kg)	8.82×10^{1}
Clay (ton)	1.959×10^{-1}		
Steel slag (ton)	1.143×10^{-1}		
Coal average (ton)	4.104×10^{-2}		
Electricity (kwh)	4.89×10^{0}		
Natural gas (ton)	2.085×10^{2}		
**Transport**			
Road (km)	1×10^{2}	Carbon dioxide (CO_2) (kg)	1.312×10^{1}
Railway (km)	2×10^{2}		
Pipeline (km)	2×10^{2}		
**Raw material preparation**			
Raw material (ton)	1.6325×10^{0}		
Coal average (ton)	4.104×10^{-2}		
Electricity (kwh)	3.22×10^{1}		
**Calcination**			
Raw material (ton)	1.6325×10^{0}	Carbon dioxide (CO_2) (kg)	1.382×10^{2}
Coal average (ton)	4.104×10^{-2}		
Electricity (kwh)	5.29×10^{2}	Clinker (ton)	1
Natural gas (ton)	2.085×10^{2}		

Table S5. Estimation of total product cost

Component	Base	
	**Conventional cement production line**	**Process Re-engineering cement line**
**Production**	1 million tons cement	1 million tons cement and 740 million m ^{3}syngas
**Service life**	20 years	
**Product Prices**	Cement: 500 CNY<br>Natural gas: 1.4 CNY/m^3<br>Syngas: 0.7 CNY/m^3	
**Fixed investment**	350 million CNY	400 million CNY (Retrofit of equipment)
**Total production cost**		
Raw materials	40.5 CNY/t	306.5 CNY/t (Increased cost of methane)
Fuel & Power	144.5 CNY/t	358.4 CNY/t (Increased cost of electricity)
Depreciation	14 CNY/t	19 CNY/t
Labor cost	13 CNY/t	13 CNY/t
Other costs	18 CNY/t	18 CNY/t
**Gross Profit**	~270 million CNY/year	~300 million CNY/year

References

1 Lee H, Jung JC, Kim H *et al*. *Catal Lett* 2008; **124**: 364-8.

2 ISO. ISO/TS 14067:2013 Greenhouse gases-carbon footprint of products-requirements and guidelines for quantification and communication.

3 Cavalett O, Watanabe MD, Voldsund M *et al*. *Nat Sustain* 2024; **7**: 568-80.
